# Electroacupuncture Ameliorates Tibial Fracture-Induced Cognitive Dysfunction by Elevating *α*7nAChR Expression and Suppressing Mast Cell Degranulation in the Hippocampus of Rats

**DOI:** 10.1155/2022/3182220

**Published:** 2022-04-12

**Authors:** Yudi Zhou, Cheng Hu, Chenlu Mao, Sha Li, Yaomei Cui, Yanning Qian

**Affiliations:** ^1^Department of Anesthesiology, The First Affiliated Hospital of Nanjing Medical University, Nanjing, China; ^2^Department of Anesthesiology, Jiangsu Province Hospital of Chinese Medicine, Affiliated Hospital of Nanjing University of Chinese Medicine, Nanjing, Jiangsu, China; ^3^Affiliated Hospital of Nanjing University of Chinese Medicine, Nanjing, Jiangsu, China

## Abstract

Intracerebral neuroinflammation, closely related to brain mast cell (MC) activation, performs an integral function in the pathogenic process of postoperative cognitive dysfunction (POCD). In addition to regulating cognitive activities, the alpha-7-nicotinic acetylcholine receptor (*α*7nAChR) engages in the progression of cognitive deficiency. In this research, we aimed to investigate how electroacupuncture (EA) affects the cognitive function in rats after tibial fracture surgery to determine whether the underlying mechanism involves the inhibition of hippocampal MC degranulation via *α*7nAChR. A rat model of tibial fracture surgery for inducing POCD was developed and subjected to treatment with EA or the *α*7nAChR antagonist *α*-bungarotoxin (*α*-BGT) and the *α*7nAChR agonist PHA-543613. The spatial memory tasks in the Morris Water Maze (MWM) test showed that both EA and PHA-543613-treated rats performed significantly better than untreated rats, with reduced escape latency and increased frequency of passage through the platform. However, EA and PHA-543613 intervention decreased the protein and mRNA levels of High-mobility group box-1(HMGB-1) and proinflammatory cytokines tumor necrosis factor-*α* (TNF-*α*), interleukin-1*β* (IL-1*β*) in the serum and hippocampus, respectively, by upregulating *α*7nAChR in the hippocampus. Furthermore, EA and PHA-543613 pretreatment reduced the number of activated MCs and suppressed neuronal apoptosis after tibial fracture surgery in the hippocampal CA1 regions, which was reversed by *α*-BGT. The findings indicated that EA pretreatment ameliorated POCD after tibial fracture surgery in rats by inhibiting brain MC activation and neuroinflammation mediated by the *α*7nAChR-dependent cholinergic anti-inflammatory system.

## 1. Introduction

Postoperative cognitive dysfunction (POCD), which encompasses acute delirium and long-term cognitive dysfunction, is a prevalent side effect among aged patients who have undergone a surgical operation, especially after major elective surgeries such as cardiac surgery and hip fracture repair[1, 2]. In patients aged more than 65 years old, the incidence of POCD is considerably higher at 10–20%, which is also associated with a 30-day mortality of 7–10% [3].Given the high rate of mortality in older patients with cognitive dysfunction, it has become a major health challenge owing to the rapid increase in the aging population [4]. Therefore, it is vital to investigate possible preventative or therapeutic strategies for POCD.

The mechanisms underlying POCD pathogenesis have not been comprehensively elucidated. Increasing evidence suggests that neuroinflammation plays an instrumental role in the progression of cognitive dysfunction [5]. A previous study revealed a mechanistic association between neuroinflammation and postoperative cognitive dysfunction in animal model [6]. The hippocampus is closely related to cognitive function, and numerous studies have reported that hippocampal neuroinflammation is involved in neurocognitive disorder [7, 8]. Therefore, the inhibition of neuroinflammation may be a possible treatment strategy against cognitive dysfunction, at least to a certain extent. Cholinergic anti-inflammatory pathways perform integral function in modulating inflammation. These pathways are mainly characterized by the interactions between the alpha-7 nicotinic acetylcholine receptors (*α*7nAChR) on immune cells and the vagus nerve, which result in the suppression of proinflammatory cytokine release, thereby preventing excessive inflammation [9, 10]. Activation of *α*7nAChR exerts significant anti-inflammatory effects on neuroinflammation. *α*7nAChR reduces neuroinflammation, thereby serving as a potential therapeutic target [11, 12].

Acupuncture is a traditional Chinese medicine (TCM) therapeutic technique that includes the insertion of sharp, tiny needles into particular sites on the body to reestablish normal homeostasis and heal various illnesses [13]. Electroacupuncture (EA), which combines electrical stimulation and acupuncture, is effective in reducing cognitive impairment as well as increasing learning and memory function in both animal and human studies [14, 15]. Earlier research has indicated that EA pretreatment has a protective effect against cerebral ischemic injury [16, 17]. Acupuncture reduces cognitive impairments by activating the *α*7nAChR-mediated anti-inflammatory pathways [18]. These studies suggest that EA therapy upregulates *α*7nAChR, which in turn exerts beneficial effects against neurocognitive disorder.

The interplay between glial cells and neurons has been the subject of several postoperative delirium-focused investigations; however, only a limited number of studies have examined the role of brain mast cells (MCs) in cognitive disturbance. The “first responders” to neuroinflammation are precisely the MCs in the brain. An increase in the activation of MCs, accompanied by the activation of astrocytes, the release of inflammatory mediators, and the progression of cognitive impairments has been observed postoperatively [19]. Activated MCs led to inflammation of the central nervous system (CNS) and cognitive defects [20]. Furthermore, brain MCs are involved in the proinflammatory effects of high-mobility group box-1 (HMGB-1) on the CNS. Additionally, HMGB-1 caused an increase in the activation of brain MCs in the hippocampal CA1 area, resulting in a disruption of the blood–brain barrier [21]. Nevertheless, the effects of EA activation on brain MCs, as well as the possible function of *α*7nAChRs, have only been described in a few studies. Therefore, in the present study, we hypothesized that EA pretreatment suppressed the production of proinflammatory cytokines and the activation of MCs in the hippocampus through *α*7nAChRs. We tested this hypothesis in adult rats with tibial fracture.

## 2. Materials and Methods

### 2.1. Animals

One hundred and twenty adult male Sprague–Dawley rats (250–300 g in weight) were obtained from the Experimental Animal Center of Nanjing University of TCM (Nanjing, Jiangsu, China). All animal care and experimental protocols abided by the US National Institutes of Health's Guide for the Care and Use of Laboratory Animals.

### 2.2. Groups and Drug Treatment

**Figure 1:**
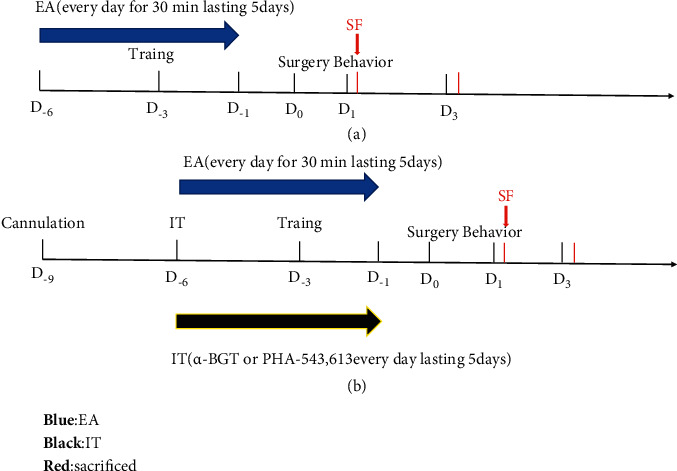
The schematic diagram for experimental procedures. (a) EA pretreatment for consecutive 5 days and tibial fracture surgery 24 h after the last EA treatment; the rats were sacrificed after the Morris water maze test on day 1 and 3 postoperatively. (b) Rats in the *α*-BGT + EA + Op group received intracerebroventricular injection of *α*-BGT (0.5 *µ*g/kg dissolved in saline 10 *µ*l,203980, Sigma, USA) for 30 min before the onset of EA for 5 days; rats in the PHA-543613+Op group received intraperitoneal injection of PHA-543613 (1.0 mg/kg dissolved in saline 1 ml, PZ0135, Sigma, USA) once a day for 5 days [22]. Rats were sacrificed after the Morris water maze test on day 1 and 3 postoperatively.

Figure 1 depicts a schematic representation of the experimental procedures. To assess the mechanism of *α*7nAChR in inhibiting MCs activation and the cognitive improvement induced by EA pretreatment after tibial fracture surgery, 120 rats were classified into five groups in a random manner, namely control (rats did not undergo surgery), operation (tibial fracture surgery, Op), EA + Op, alpha-bungarotoxin (*α*-BGT)+EA + Op, and PHA-543613+Op groups. For the rats belong to the EA + Op group, EA was administered for 5 consecutive days and subjected to additional surgical intervention 1 day following the final EA pretreatment. Each group was further classified into two subgroups based on the two-time points that they were selected (1 and 3 days postoperatively) (Figures 1(a) and 1(b)).

### 2.3. Tibial Fracture Surgery

POCD model was induced by tibial fracture surgery as reported previously [20]. Briefly, rats were anesthetized using isoflurane (2.1%), and buprenorphine (0.1 mg/kg). They were subcutaneously injected to induce analgesia. Following the loss of the righting reflex, open tibial fracture surgery of the left posterior paw was conducted in aseptic conditions, with intramedullary fixation. The left posterior paws of the surgical animals were shaved, followed by disinfection using povidone-iodine before being used. A 7-mm G pin was introduced into the intramedullary canal by performing a median incision, followed by the removal of the periosteum and the performing of the osteotomy. Eventually, the wound area was irrigated, and the skin was subjected to suturing. For the animals belong to the control group, no surgical intervention was provided. However, equivalent dosages of isoflurane (2.1%) and buprenorphine (0.1 mg/kg) were administered. During surgery, the temperature was continued to maintain at 37 ± 0.5°C with the heating blanket.

### 2.4. EA Pretreatment

The Baihui acupoint (GV 20) was selected for EA pretreatment in view of the fact that it lies at the point where the sagittal midline, as well as the line connecting the two ears [17]. Specifically, the rats were anesthetized, and stimulation of the GV20 was performed for half an hour at a 2/15 Hz frequency and 1 mA intensity with the aid of an electronic acupuncture treatment device (Model No. SDZ-V, Suzhou Medical Appliances Co., Ltd., Suzhou, China). A tibial fracture surgery was performed 1 day following the final EA treatment on the rats, which had been exposed to EA for a total of 5 consecutive days prior to the operation.

### 2.5. Intracerebroventricular Cannula Implantation

After being anesthetized, rats were placed in the stereotaxic device (RWD, Shenzhen, China) for the purpose of injecting medicines into the intracerebroventricular region. The below-mentioned precise locations were used to insert a guiding cannula with 21-gauge stainless steel into the right lateral ventricle: 3.7 mm ventral, 1.5 mm right lateral, and 0.8 mm posterior to the bregma. Subsequently, the guiding cannula was fastened with dental cement and affixed to the skull with stainless steel screws that were implanted surgically. In the course of their recovery period of at least 3 days, the rats were kept separately for subsequent use in the following experiments. The guide cannula was checked daily to keep rats familiarized with the investigators. For the purpose of administering the drug solution at the desired pace, a 29-gauge injector cannula that had been attached to a microsyringe pump via a PE-20 tubing was loaded using the solution at a rate of 0.5 *μ*L/min.

### 2.6. Morris Water Maze Test

As described in an earlier study [23], training of the rats was conducted on the Morris water maze four times a day for 3 days prior to surgery, and their cognitive performance was assessed on days one and three postoperatively. The navigation positioning assessment was the first of these tests. We positioned a platform that was transparent within any of the four quadrants and the top of the platform was set at 1 centimeter under the water surface. A platform was set up in the farthest quadrant from the rats, and they can discover and climb on top of it. The length of this period was reported as the escape latency. If the rat fails to locate the platform, the escape delay was subjected to a measurement at 90 seconds. The average escape latency of each rat was calculated by means of taking the mean of the four quadrants. The space exploration assessment was the second of the three tests. Then, we removed the platform from the pool and placed the rats in water in the opposite quadrant of the former test quadrant. The evaluator monitored each rat's swimming pattern for 90 seconds and counted the number of times the rats crossed over the initial platform location (platform crossing number).

### 2.7. Eenzyme-linked Immunosorbent Assay (ELISA)

We harvested blood samples from the femoral veins of rats on days 1 and 3 postoperatively. After centrifuging blood at 3000 revolutions per minute for 15 minutes at a temperature of 4°C, the supernatant was extracted and kept at a freezing temperature of −80°C. The levels of TNF-*α*, IL-1*β*, and HMGB-1 in serum were assessed with the aid of enzyme-linked immunosorbent assay (ELISA) kits (Huamei bio, China).

### 2.8. qRT-PCR

TRIzol reagent (Invitrogen, USA) was employed for the purpose of extracting total RNA from hippocampi, and the extracted RNA was subsequently reverse-transcribed with the aid of the Transcription First Strand cDNA Synthesis Kit (Roche, Switzerland). Real-time polymerase chain reaction (PCR) amplification was performed based on an ABI7500 Real-time PCR Detection System (Foster City, CA, USA) with SYBR Green master mix (Applied Biosystems, USA). The GAPDH gene control values were employed to normalize the relative mRNA levels and were derived by means of the comparative cycle threshold technique.

Listed below is a list of primer sequences:

  TNF-*α*: forward, 5′-CCGAGATGTGGAACTGGCAGAG-3′;
  reverse, 5′-CCACGAGCAGGAATGAGAAGAGG-3′.
  IL-1*β*: forward, 5′-CAGCTTTCGACAGTGAGGAGA-3′;
  reverse, 5′-TTGTCGAGATGCTGCTGTGA-3′.
  HMGB-1: forward, 5′-AAGGCTGACAAGGCTCGTTATGAA-3′;
  reverse, 5′-ATGCGGATTGGGCTGGTTTCTT-3′,
  GAPDH: forward, 5′-AGTGCCAGCCTCGTCTCATA-3';
  reverse, 5′-GGTAACCAGGCGTCCGATAC-3′.

### 2.9. Western Blotting Analysis of *α*7nAChR

The extraction of hippocampal proteins was performed with the assistance of ice-cold RIPA lysis buffer (Beyotime, Shanghai, China), which includes a protease inhibitor (Beyotime, Shanghai, China) and a phosphatase inhibitor (Boster, Wuhan, China). As previously reported, we employed the Western blot technique to assess the expression of the *α*7nAChR subunit [22]. The membranes were incubated with primary antibodies against rabbit *α*7nAChR (Abcam, ab216485, 1 : 1000) and against mouse *ß*-actin (Boster, Wuhan, China, BM0627, 1 : 500) then incubated with homologous secondary antibodies. Enhanced chemiluminescence (Bio-Rad) was employed with the goal of visualizing the protein bands on the membranes. Subsequently, the Image Lab program (Bio-Rad, Richmond, CA, USA) was used to scan the protein bands in each group and determine their relative density in each group. With the aid of the NIH Image J program (Bethesda, MD, USA), we successfully determined the intensity of the bands.

### 2.10. Toluidine Blue Staining and Cell Counting

Hippocampal sections collected from different groups were stained with 0.05% toluidine blue (TB). Subsequently, the slides were immersed in TB solution for 30 min, followed by rinse twice using distilled water, desiccation using a sequence of increasing dosages of ethanol, and placing in butyl acetate ester. The samples were embedded in the Eukitt® mounting medium under coverslips and left to air-dry throughout the night. Listed below were the degranulation criteria: a shape that has been warped, fuzzy appearance, the disappearance of purple stains, and numerous granules seen in the proximity of the cells. A light microscope (Leica Microsystems, Wetzlar, Germany) was used to visualize the CA1 region of three slices of each rat at 200× magnification (high-power field), yielding a total of 15 images per rat. The NIH Image J program (Bethesda, MD, USA) was employed to obtain the number of MC for each image (0.74-mm^2^ frame), which were then averaged and transformed into cells/mm^2^. Notably, the researcher performed the MCs counting in a blinded manner.

### 2.11. TUNEL Staining

We conducted quantitative terminal deoxynucleotidyl transferase dUTP Nick end labeling (TUNEL) staining as reported earlier for detecting the apoptosis of neuronal cells in the hippocampal tissue [22]. An optical microscope (Leica Microsystems, Wetzlar, Germany) was utilized to visualize the CA1 region of three slices of each rat, yielding a total of 15 images per rat being taken at 200× magnification (high-power field). Subsequently, the NIH Image J program (Bethesda, MD, USA) was employed for the purpose of determining the proportion of apoptotic cells in each image (0.74 mm^2^ frame), which was subsequently averaged before conversion to cells/mm^2^. Similarly, the researcher performed this procedure in a blinded manner.

## 3. Statistical Analysis

The GraphPad Prism software (version 5, GraphPad Software, San Diego, CA) was employed to perform analyses of statistical data. The results were presented as the mean ± standard error of the mean (SEM). The analyses of data were conducted by one-way or two-way analysis of variance (ANOVA), followed by the Newman–Keuls posthoc test wherever appropriate. The criterion for determining statistical significance was set at *P* < 0.05.

## 4. Results

### 4.1. EA Improved Tibial Fracture-induced Cognitive Impairment

**Figure 2:**
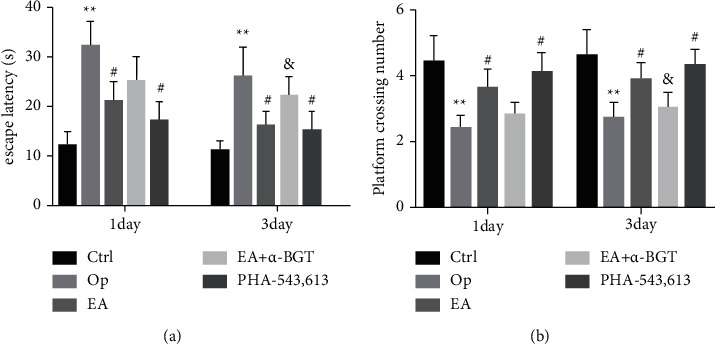
EA improved tibial fracture-induced cognitive impairment (a) The escape latency in rats was measured using the Morris water maze test 1 and 3 days after tibial fracture surgery. (b) The number of crossing the platform was measured using the Morris water maze 1 and 3 days after tibial fracture surgery. Graphs show the mean ± S.E.M. (*n* = 12). ^∗∗^*P* < 0.01 versus the control group; ^#^*P* < 0.05 versus the Op group; ^&^*P* < 0.05 versus the EA group.

To investigate the impacts of EA, *α*-BGT, and PHA-543613 pretreatment on the memory and learning function of rats undergoing surgery for tibial fracture, we employed the Morris water maze to validate the findings on days 1 and 3 following a surgical operation. As illustrated in Figures 2(a) and 2(b), on days 1 and 3 following surgery, the rats in the Op group exhibited a considerably longer escape latency time (*P* < 0.01) as well as a substantially reduced number of platform crossings (*P* < 0.01) in contrast with the control rats. Such results indicate that tibial fracture surgery can damage the memory and learning function of adult rats postoperatively. In the EA- and PHA-543613-treated groups, the latency time was substantially shortened, and the platform crossing number was considerably elevated in contrast with the Op group (day 1: *P* < 0.05, day 3: *P* < 0.05). As opposed to the rats in the EA subgroup, the rats who received EA+*α*-BGT displayed longer latency time as well as lower platform crossing numbers(day 3: *P* < 0.05). However, no differences were identified between the EA and EA+*α*-BGT groups (day 1: *P* > 0.05).

### 4.2. EA Attenuated Neuroinflammation and Peripheral Inflammation in a Rat after Tibial Fracture Surgery

**Figure 3:**
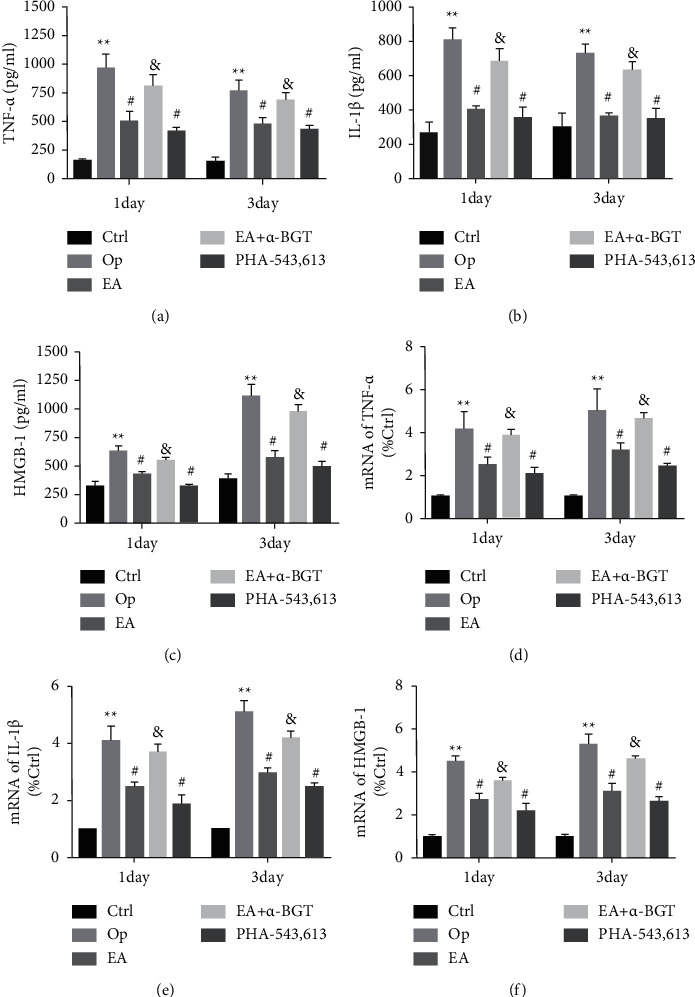
EA attenuated neuroinflammation and peripheral inflammation in rats after tibial fracture surgery. The serum TNF-*α*, IL-1*β*, and HMGB-1 levels were measured using ELISA on day 1 and 3 postoperatively (a–c). The mRNA expression of TNF-*α*, IL-1*β*, and HMGB-1 in the hippocampus on day 1 and 3 postoperatively was determined using real-time PCR (d–f). Graphs show the mean ± S.E.M. (*n* = 6). ^∗∗^*P* < 0.01 versus the control group; ^#^*P* < 0.05 versus the Op group; ^&^*P* < 0.05 versus the EA group.

The proinflammatory cytokines levels in the serum of rats, such as IL-1*β*, HMGB-1, and TNF-*α*, were assessed after tibial fracture surgery on days 1 and 3. In the Op group, the IL-1*β*, HMGB-1, and TNF-*α* levels were substantially elevated as opposed to those in the control group (day 1: *P* < 0.01, day 3: *P* < 0.01). Their concentrations were significantly downregulated in both EA and PHA-543613 groups in contrast with the Op group (day 1: *P* < 0.05, day 3: *P* < 0.05). Moreover, the expression levels of these proinflammatory markers were elevated in the EA+*α*-BGT as opposed to the EA group (day 1: *P* < 0.05, day 3: *P* < 0.05). Nonetheless, they did not differ obviously from those of the Op group (Figures 3(a)–3(c)).

For the purpose of assessing the influence of EA on neuroinflammation, we performed qRT-PCR to determine the expression levels of *IL-1β, HMGB-1*, and *TNF-α* mRNA in the hippocampus of rats. Data analysis revealed that the expression levels of *IL-1β, HMGB-1*, and *TNF-α* mRNA in the Op group were elevated postoperatively (day 1: *P* < 0.01, day 3: *P* < 0.01) in contrast with those in the control group. Moreover, the expression levels of hippocampal *IL-1β, HMGB-1*, and *TNF-α* mRNA were remarkably inhibited in the EA- and PHA-543613-treated groups compared with the Op group at the same time points (day 1: *P* < 0.05, day 3: *P* < 0.05). The expression levels of *IL-1β, HMGB-1*, and *TNF-α* were considerably elevated in the EA+*α*-BGT group compared with the EA group (day 1: *P* < 0.05, day 3: *P* < 0.05). No significant difference was identified in the expression levels of *IL-1β, HMGB-1*, and *TNF-α* mRNA on days 1 and 3 postoperatively between the Op and EA+*α*-BGT groups (*P* > 0.05) (Figures 3(e) and 3(f)). Therefore, EA downregulated expression levels of *IL-1β, HMGB-1*, and *TNF-α* in the serum and hippocampus postoperatively, indicating that it can improve cognitive function by decreasing the levels of these inflammatory cytokines.

### 4.3. EA Inhibited MC Degranulation Associated with *α*7nAChR Activation in the Hippocampus

**Figure 4:**
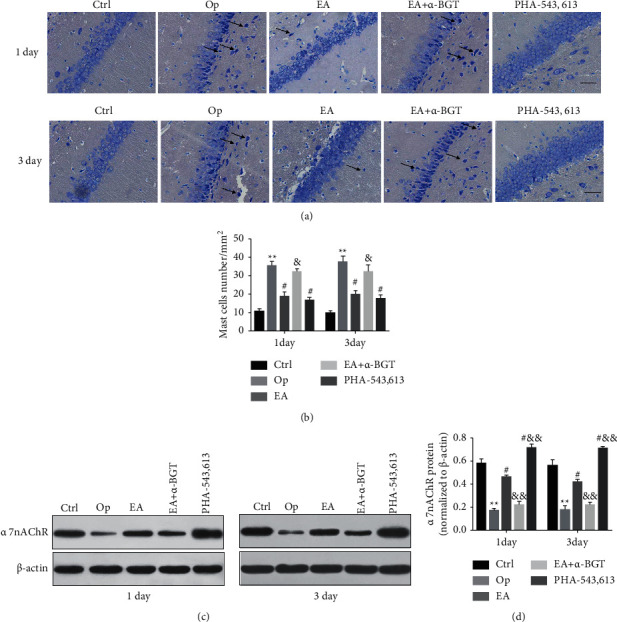
EA inhibited MC degranulation associated with *α*7nAChR activation in the hippocampus. Hippocampal sections (10 *µ*m) were prepared 1 and 3 days after surgery. (a) Brain mast cells were stained with toluidine blue (TB). (b) Quantification of mast cells in the hippocampal CA1 region on day 1 and 3 postoperatively. (c) Western blot analysis of *α*7nAChR protein expression in the hippocampus on day 1 and 3 postoperatively. (d) Quantified and normalized to *ß*-actin levels. Graphs show the mean ± S.E.M. (*n* = 4). ^∗∗^*P* < 0.01 versus the control group; ^#^*P* < 0.05 versus the Op group; and ^&&^*P* < 0.01 or ^&^*P* < 0.05versus the EA group, scale bar = 25 *µ*m.

Brain MCs were quantified in TB-stained sections. The count of MCs in the hippocampal CA1 area in the Op group was remarkably elevated on day 1 (*P* < 0.01) and day 3 (*P* < 0.01) postoperatively as opposed to that in the controls. The count of MCs in the EA- and PHA-543613-treated groups decreased when compared with that in the Op group (day 1: *P* < 0.05, day 3: *P* < 0.05). A considerable difference was observed in the count of MCs between the EA and EA+*α*-BGT groups (day 1: *P* < 0.05, day 3: *P* < 0.05) (Figures 4(a) and 4(b)).

An investigation on the protein expression levels of the *α*7nAChR in the hippocampus was carried out using Western blotting. The levels of *α*7nAChR protein were substantially reduced in the hippocampus of the Op group as opposed to the controls on days 1 and 3 postoperatively (*P* < 0.01). Nonetheless, the expression levels of the hippocampal *α*7nAChR protein in the EA- and PHA-543613-treated groups were remarkably elevated on day 1 and 3 postoperatively (*P* < 0.05), substantially lowered in the EA+*α*-BGT group relative to the EA group (*P* < 0.01) and significantly elevated in the PHA-543613 group (*P* < 0.01). Furthermore, the protein expression level of *α*7nAChR on days 1 and 3 postoperatively between the Op and EA+*α*-BGT groups was found to have no significant differences (*P* > 0.05) (Figure 4(c)–4(d)). According to these findings, it was hypothesized that *α*7nAChR activation was involved in the prevention of MCs degranulation related to the anti-POCD role of EA.

### 4.4. EA Inhibited Tibial Fracture-Induced Apoptosis in the Hippocampus

**Figure 5:**
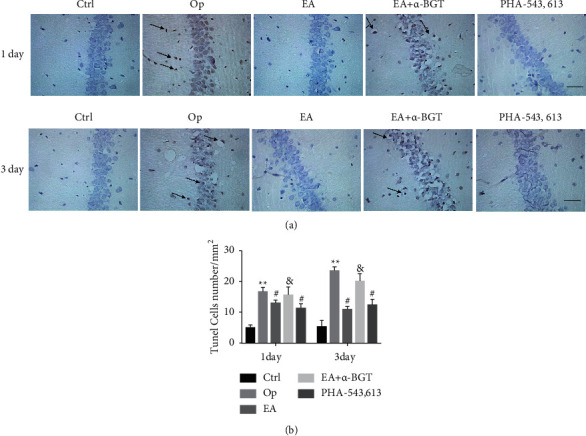
EA inhibited tibial fracture-induced apoptosis in the hippocampus. Hippocampal sections (10 µm) were prepared 1 and 3 days after surgery. (a) The apoptosis of neuronal cells was detected using TUNEL. (b) Quantification of apoptotic cells in the hippocampal CA1 region on day 1 and 3 postoperatively. Graphs show the mean ± S.E.M (*n* = 4). ^∗∗^*P* < 0.01 versus the control group; ^#^*P* < 0.05 versus the Op group; ^&^*P* < 0.05 versus the EA group; scale bar = 25 *µ*m.

On days 1 and 3 following surgery, the count of TUNEL-positive cells within the hippocampus CA1 region was remarkably elevated in the Op group in contrast with that in the control group (*P* < 0.01) and reduced in the groups treated with EA- and PHA-543613 (day 1: *P* < 0.05, day 3: *P* < 0.05). When comparing the EA and EA+*α*- BGT groups, significant differences were observed in the count of TUNEL-positive cells (day 1: *P* < 0.05, day 3: *P* < 0.05). The above data revealed that pretreatment with EA reduced apoptosis in the hippocampus following tibial fracture surgical procedure and that the protective function of EA pretreatment was reversed by the *α*7nAChR antagonist *α*-BGT (Figures 5(a) and 5(b)).

## 5. Discussion

MCs play a pivotal role in the pathophysiology of CNS-related diseases such as stroke, neurodegenerative disease, brain tumors, and traumatic brain injury [24]. Furthermore, the production of MC activation-dependent inflammatory mediators augments neuroinflammation. In this study, we demonstrated that brain MCs were involved in tibial fracture-induced cognitive impairment and that EA-activated cholinergic neuroimmune interaction via *α*7nAChRs on brain MCs, which perform an integral function in controlling neuroinflammation and peripheral inflammation. Additionally, EA pretreatment improved tibial fracture-induced cognitive impairment in rats.

A previous study suggested that tibial fracture surgery could effectively simulate the conditions of POCD in rats [25]. The Morris water maze test is a frequently utilized tool to assess spatial memory and learning in mice, and it is a highly reliable method of cognitive assessment [26]. In this study, the escape platform was stable during the test. The hippocampus-dependent spatial reference memory and learning function were assessed in the rats. Our results demonstrated that the cognitive ability of rats was decreased postoperatively; however, pretreatment using EA or the *α*7nAChR agonist PHA-543613 partially reversed cognitive impairment, whereas the effect of EA on cognitive improvement was partially reversed by *α*-BGT, a selective *α*7nAChR antagonist. This indicated that cognitive dysfunction was induced successfully in our animal model and that *α*7nAChR might mediate the cognitive improvement induced by EA in rats after tibial fracture surgery.

Sustained neuroinflammation is implicated in the progression of cognitive impairment [27]. Increasing evidence suggests that postoperative trauma causes central and systemic inflammation, resulting in cognitive dysfunction. Proinflammatory cytokines, such as IL-1*β* and TNF-*α*, contribute to the initiation of neuroinflammation. Studies have reported that high levels of IL-1*β* and TNF-*α* in plasma and brain tissues are associated with POCD [28, 29]. According to recent studies, high levels of HMGB-1 were associated with postoperative impairment of memory function in rats [30, 31]. Our results suggested that on days 1 and 3 following surgery, the levels of mRNA expression of *HMGB-1, IL-1β,* and *TNF-α* in the hippocampus, and these proinflammatory cytokines in the serum significantly increased compared to those in the control group. Nonetheless, pretreatment with EA or the *α*7nAChR agonist PHA-543613 reversed the surgery-induced increase in HMGB-1, IL-1*β*, and TNF-*α* production. Furthermore, rats administered *α*-BGT did not show any anti-inflammatory benefits after receiving EA as a pretreatment. These findings indicated that *α*7nAChR might mediate decreased systemic and central inflammation induced by EA.

*α*7nAChR is a cholinergic-dependent signaling molecule that is implicated in various CNS disorders, such as Alzheimer's disease, schizophrenia, anxiety, depression, and Parkinson's disease [32]. *α*7nAChR is strongly expressed in the brain, particularly in the hippocampus. Furthermore, inhibition of *α*7nAChR by an *α*7nAChR antagonist exacerbated cognitive disorders in postoperative mice under general anesthesia [33]. However, trauma-induced neuroinflammation is prevented by preoperative treatment with *α*7nAChR agonists, thus ameliorating cognitive decline [34]. PHA-543613 is a selective *α*7nAChR agonist. A previous study demonstrated that pretreatment with PHA-543613 successfully reversed scopolamine-induced cognitive decline [12]. These findings suggested that cholinergic dysfunction is a pathogenic mechanism underlying POCD, which was in agreement with our findings that hippocampal *α*7nAChR protein levels were reduced in rats following tibial fracture surgery on days 1 and 3. Our study also demonstrated that EA and PHA-543613 pretreatment had a similar effect in reversing the reduction in *α*7AChR expression in rats postoperatively. In contrast, the increase in *α*7AChR protein expression induced by EA was reversed by the selective *α*7nAChR antagonist *α*-BGT. These findings suggested that *α*7nAChR activation is a critical mediator of the neuroprotective effects of EA.

In neurodegenerative disorders, MC activity is an early sign that often precedes neuroinflammation and neuronal death [35]. Once activated, brain MCs can rapidly release inflammatory chemicals that activate microglia, induce apoptosis of neurons, and disrupt the blood–brain barrier, thereby playing a critical role in the pathogenesis of neuroinflammation [36]. A recent study reported that *α*7nAChR might serve as an immunomodulator and perform a distinct function in MCs. Additionally, mucosal MCs (MMCs) were shown to be the primary cause of disease in a food allergy model. Moreover, the cholinergic neuroimmune interactions facilitated by *α*7nAChRs on MMCs were found to be critical for modulating pathological immune activation and restoring intestinal homeostasis [37]. In our study, EA and PHA-543613 pretreatment significantly lowered the proportion of activated MCs in the hippocampus; however, *α*-BGT reversed the EA-induced inhibition of activated MC to a certain extent. These findings indicate that EA inhibits hippocampal MC activation through *α*7nAChR.

Apoptosis, which is a form of programmed cell death, performs an instrumental function in neurodegenerative diseases [38]. A previous study illustrated that EA at GV29 and GV20 in rats significantly decreased the rate of neuronal apoptosis in the hippocampus, thereby effectively alleviating cognitive impairment [39]. In our study, we found a small number of TUNEL-positive neuronal cells in the EA- and PHA-543613-treated groups as opposed to the surgery group. Apoptosis was not inhibited by EA pretreatment in rats suffering from tibial fractures who were administered *α*-BGT. Therefore, we hypothesized that cholinergic signaling is essential in the neuroprotective effects of EA on anti-apoptosis.

To the best of our knowledge, the present study is the first to examine the immune-regulatory effects of EA at the Baihui (GV20) acupoint based on the inherent correlation between brain MCs and *α*7nAChR after tibial fracture surgery. However, there are some limitations in our study. First, we performed adult rats of POCD model in this study, aged rats of tibial fracture surgery should be further investigated. Second, in the future, mice with MC gene deletion or *α*7nAChR knockout could be used to further confirm the mechanism of EA in hippocampal MC *α*7nAChR improvement in mice after tibial fracture surgery. Third, not only Baihui (GV20) acupoint, but other acupoints should be studied. Finally, in recent years, acupuncture combined with ancient classical formulas medicine have been widely used in the treatment of chronic and critical diseases; so, in the follow up research, we will investigate the influence of EA combined with ancient classical formulas on the inherent correlation between brain MCs and *α*7nAChR after tibial fracture surgery.

In conclusion, the present study demonstrated that EA significantly ameliorated surgery-induced cognitive impairment by inhibiting the activation of brain MCs and neuroinflammation mediated by the *α*7nAChR-dependent cholinergic anti-inflammatory system.

## Data Availability

The data used to support the findings of this study were included in the article. All the authors declare that all the data in the manuscript were obtained by our experiments and the data are true and effective in the manuscript. The datasets used in the present manuscript are available from the corresponding author on reasonable request.

## Abbreviations

MC: Mast cell
POCD: Postoperative cognitive dysfunction
*α*7nAChR: Alpha-7-nicotinic acetylcholine receptor
EA: Electroacupuncture
*α*-BGT: Alpha-bungarotoxin
MWM: Morris water maze
TNF-*α*: Tumor necrosis factor-*α*
IL-1*β*: Interleukin-1*β*
HMGB-1: High-mobility group box-1
TCM: Traditional Chinese medicine
CNS: Central nervous system
NIH: National Institutes of Health
ELISA: Enzyme-linked immunosorbent assay
RT-PCR: Real-time polymerase chain reaction
TB: Toluidine blue
TUNEL: Terminal-deoxynucleotidyl transferase dUTP nick end labeling
MMCs: Mucosal mast cells.

## References

[1] Avelino-Silva T. J., Campora F., Curiati J. A. E., Jacob-Filho W., (2017). Association between delirium superimposed on dementia and mortality in hospitalized older adults: a prospective cohort study. *PLoS Medicine*.

[2] Jin Z., Hu J., Ma D. (2020). Postoperative delirium: perioperative assessment, risk reduction, and management. *British Journal of Anaesthesia*.

[3] Daiello L. A., Racine A. M., Marcantonio E. R. (2019). Postoperative delirium and postoperative cognitive dysfunction: overlap and divergence. *Anesthesiology*.

[4] Evered L., Silbert B., Knopman D. S. (2018). Recommendations for the nomenclature of cognitive changes associated with anaesthesia and surgery-2018. *British Journal of Anaesthesia*.

[5] Subramaniyan S., Terrando N. (2019). Neuroinflammation and perioperative neurocognitive disorders. *Anesthesia & Analgesia*.

[6] Feng X., Uchida Y., Koch L. (2017). Exercise prevents enhanced postoperative neuroinflammation and cognitive decline and rectifies the gut microbiome in a rat model of metabolic syndrome. *Frontiers in Immunology*.

[7] Liu Q., Sun Y. M., Huang H. (2021). Sirtuin 3 protects against anesthesia/surgery-induced cognitive decline in aged mice by suppressing hippocampal neuroinflammation. *Journal of Neuroinflammation*.

[8] Feng P. P., Deng P., Liu L. H. (2017). Electroacupuncture alleviates postoperative cognitive dysfunction in aged rats by inhibiting hippocampal neuroinflammation activated via microglia/TLRs pathway. *Evidence-based Complementary and Alternative Medicine*.

[9] Frasch M. G., Szynkaruk M., Prout A. P. (2016). Decreased neuroinflammation correlates to higher vagus nerve activity fluctuations in near-term ovine fetuses: a case for the afferent cholinergic anti-inflammatory pathway?. *Neuroinflammation*.

[10] Song C., Shi J., Xu J. (2021). Post-transcriptional regulation of *α*7 nAChR expression by miR-98-5p modulates cognition and neuroinflammation in an animal model of Alzheimer’s disease. *Federation of American Societies for Experimental Biology Journal*.

[11] Adigh-Eteghad S., Talebi M., Mahmoudi J. (2015). Selective activation of *α*7 nicotinic acetylcholine receptor by PHA-543613 improves A*β*25-35-mediated cognitive deficits in mice. *Neuroscience*.

[12] Bruszt N., Bali Z. K., Tadepalli S. A., Nagy L. V., Hernádi I. (2021). Potentiation of cognitive enhancer effects of Alzheimer’s disease medication memantine by alpha7 nicotinic acetylcholine receptor agonist PHA-543613 in the Morris water maze task. *Psychopharmacology (Berl)*.

[13] Zhao L., Chen J., Liu C. Z. (2012). A review of acupoint specificity research in China: status quo and prospects. *Evidence-based Complementary and Alternative Medicine*.

[14] Sun L., Yong Y., Wei P. (2022). Electroacupuncture ameliorates postoperative cognitive dysfunction and associated neuroinflammation via NLRP3 signal inhibition in aged mice. *CNS Neuroscience and Therapeutics*.

[15] Liu P. R., Zhou Y., Zhang Y., Diao S. (2017). Electroacupuncture alleviates surgery-induced cognitive dysfunction by increasing *α*7-nAChR expression and inhibiting inflammatory pathway in aged rats. *Neuroscience Letters*.

[16] Wu Z. Q., Cui S. Y., Zhu L., Zou Z. Q. (2016). Study on the mechanism of mTOR-mediated autophagy during electroacupuncture pretreatment against cerebral ischemic injury. *Evidence-based Complementary and Alternative Medicine*.

[17] Mao C., Hu C., Zhou Y. (2020). Electroacupuncture pretreatment against cerebral ischemia/reperfusion injury through mitophagy. *Evidence-based Complementary and Alternative Medicine*.

[18] Cao Y., Lin L. W. L. T. (2021). Acupuncture attenuates cognitive deficits through *α*7nAChR mediated anti-inflammatory pathway in chronic cerebral hypoperfusion rats. *Life Sciences*.

[19] Zhang X., Yao H., Qian Q. (2016). Cerebral mast cells participate in postoperative cognitive dysfunction by promoting astrocyte activation. *Cellular Physiology and Biochemistry*.

[20] Zhang S., Dong H., Zhang X., Li N., Sun J., Qian Y. (2016). Cerebral mast cells contribute to postoperative cognitive dysfunction by promoting blood–brain barrier disruption. *Behavioural Brain Research*.

[21] Qian Q. Q., Zhang X., Wang Y. W. (2019). Pro-inflammatory role of high-mobility group box-1 on brain mast cells via the RAGE/NF-*κ*B pathway. *Journal of Neurochemistry*.

[22] Wang Q., Wang F., Li X. (2012). Electroacupuncture pretreatment attenuates cerebral ischemic injury through *α*7 nicotinic acetylcholine receptor-mediated inhibition of high-mobility group box 1 release in rats. *Journal of Neuroinflammation*.

[23] Morris R. (1984). Developments of a water-maze procedure for studying spatial learning in the rat. *Journal of Neuroscience Methods*.

[24] Skaper S. D., Facci L., Zusso M., Giusti P. (2019). Neuroinflammation, mast cells, and glia: dangerous liaisons. *The Neuroscientist*.

[25] Feng X., Degos V., Koch L. G. (2013). Surgery results in exaggerated and persistent cognitive decline in a rat model of the Metabolic Syndrome. *Anesthesiology*.

[26] Vorhees C. V., Williams M. T. (2006). Morris water maze: procedures for assessing spatial and related forms of learning and memory. *Nature Protocols*.

[27] Granger K. T., Barnett J. H. (2021). Postoperative cognitive dysfunction: an acute approach for the development of novel treatments for neuroinflammation. *Drug Discovery Today*.

[28] Cibelli M., Terrando A. R. F. N. (2016). Role of interleukin-1beta in postoperative cognitive dysfunction. *Annals of Neurology*.

[29] Terrando N., Monaco C., Ma D. (2010). Tumor necrosis factor-alpha triggers a cytokine cascade yielding postoperative cognitive decline. *Proceedings of the National Academy of Sciences of the United States of America*.

[30] Lin F., Shan W., Zheng Y., Pan L., Zuo Z. (2021). Toll-like receptor 2 activation and up-regulation by high mobility group box-1 contribute to post-operative neuroinflammation and cognitive dysfunction in mice. *Journal of Neurochemistry*.

[31] Yu M., Huang H., Dong S., Sha H., Wei W., Liu C. (2019). High mobility group box-1 mediates hippocampal inflammation and contributes to cognitive deficits in high-fat high-fructose diet-induced obese rats. *Brain, Behavior, and Immunity*.

[32] Taly A., Corringer P. J., Guedin D., Changeux P. (2009). Nicotinic receptors: allosteric transitions and therapeutic targets in the nervous system. *Nature Reviews Drug Discovery*.

[33] Terrando N., Eriksson L. I., Ryu J. K. (2011). Resolving postoperative neuroinflammation and cognitive decline. *Annals of Neurology*.

[34] Kong F. J., Ma L. L., Zhang H. H., Zhou J. Q. (2015). Alpha 7 nicotinic acetylcholine receptor agonist GTS-21 mitigates isoflurane-induced cognitive impairment in aged rats. *Journal of Surgical Research*.

[35] Silver R., Curley J. P. (2013). Mast cells on the mind: new insights and opportunities. *Trends in Neurosciences I*.

[36] Kempuraj D., Mentor S., Thangavel R. (2019). Mast cells in stress, pain, blood-brain barrier, neuroinflammation and alzheimer’s disease. *Frontiers in Cellular Neuroscience*.

[37] Yamamoto T., Kodama T., Lee J. (2014). Anti-allergic role of cholinergic neuronal pathway via *α*7 nicotinic ACh receptors on mucosal mast cells in a murine food allergy model. *PLoS One*.

[38] Obulesu M., Lakshmi M. J. (2014). Apoptosis in Alzheimer’s disease: an understanding of the physiology, pathology and therapeutic avenues. *Neurochemical Research*.

[39] He X., Zhao S., You W. (2018). Neuroprotective effects of electroacupuncture preventive treatment in senescence-accelerated mouse prone 8 mice. *Chinese Journal of Integrative Medicine*.

